#  Molecular and Physiological Mechanisms of Membrane Receptor Systems Functioning 

**Published:** 2011

**Authors:** E.S. Severin, M.V. Savvateeva

**Affiliations:** All-Russia Research Center for Molecular Diagnostics and Therapy; Biology Faculty, Lomonosov Moscow State University

**Keywords:** molecular physiology, receptor profile, secondary messengers, targeted delivery, epigenetic diagnostics, prostate cancer

## Abstract

Molecular physiology is a new interdisciplinary field of knowledge that looks into how complicated biological systems function. The living cell is a relatively simple, but at the same time very sophisticated biological system. After the sequencing of the human genome, molecular physiology has endeavored to investigate the systems of cellular interactions at a completely new level based on knowledge of the spatial organization and functions of receptors, their ligands, and protein-protein interactions. In recent years, the achievements in molecular physiology have centered on the study of sensor reception mechanisms and intercellular data transfer, as well as the immune system physiology, amongst other processes.

## RESEARCH

**Fig. 1:**
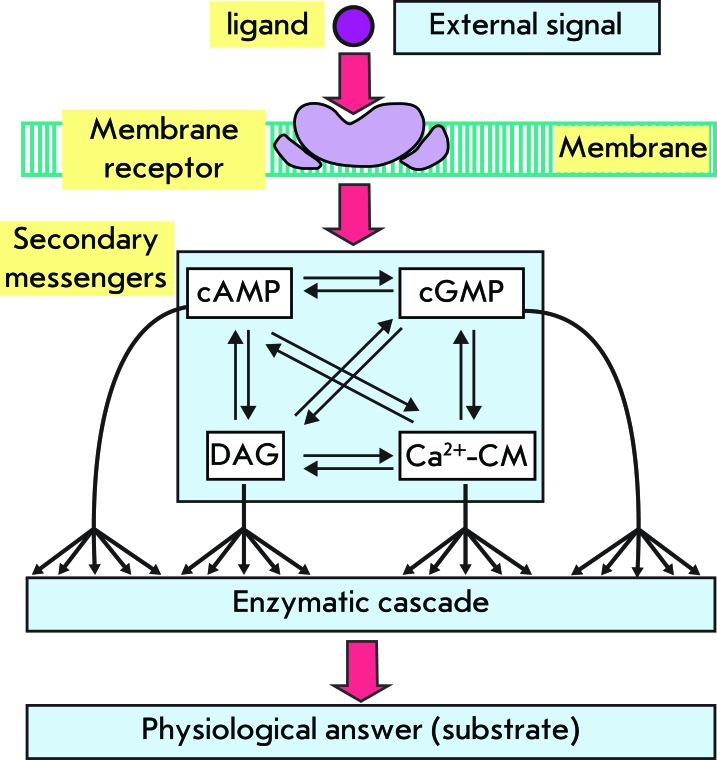
The main stages of signal transduction mediated by secondary messengers in the cell (cAMP - cyclic adenosine monophosphate, cGMP - cyclic guanosine monophosphate, DG - diacylglycerol).

When considering the functioning mechanisms of membrane receptor systems, it is necessary to first highlight the achievements in molecular physiology regarding the process regulation that occurs in the cell, as well as the intracellular transmission of hormonal signals. The concept of secondary messengers (secondary mediators) is considered today fundamental in cellular and physical-chemical biology, as well as in molecular medicine. However, towards the end of the 1950s the discovery of the first biologically active substance with signal-transduction functions – cAMP – had upended concepts regarding biochemical process regulation in the cell and the intracellular mechanisms of signal transduction. It appears that the signal molecules not able to cross through the cellular membrane interact with the specific receptors and enzyme systems located on the membrane’s external surface. Thus, through interaction with membrane receptor systems, biologically active substances determine the production of one or several secondary messengers; low-weight biologically active molecules which transmit signals on intracellular effector structures. Currently, more than 10 similar molecules have been described – they are as follows: cyclic nucleotides cAMP and cGMP; inositol exchange products – inositol phosphate (IP3); diacylglycerol (DAG), as well as Ca ^2+^ ions; polynucleotide oligoA; nitrogen monoxide (NO); arachidonic acid exchange products; and a number of other substances of lipid-origin (Fig. 1).

 It appears that key stages in signal transduction mediated by secondary messengers are common to regulation systems: agonist – receptor- effector protein- secondary messenger- modulating protein component – physiological response. The main features of secondary messengers are their universality and trigger functions. Furthermore, both various molecular structures (e.g., ion channels) and multistage cascades of enzymatic reactions can act as effectors and their regulation systems. 

In the second half of the 20 ^th^ century, not a single Nobel Prize was awarded for the outstanding achievements in research concerning this group of biologically active compounds. In various years, E. Sutherland, E. Fischer and E. Krebs, F. Gilman and M. Rodbell, F. Grignard, L. Ignarro, R. Furchgott and F. Murad et al. were awarded Nobel prizes. It should be noted that the main achievements in molecular physiology are in some way linked to cell surface receptors. Studies focusing on central nervous system neurons, neuron junctions, and neural impulse distribution have led to significant improvements in our understanding of cell physiology.

**Fig. 2:**
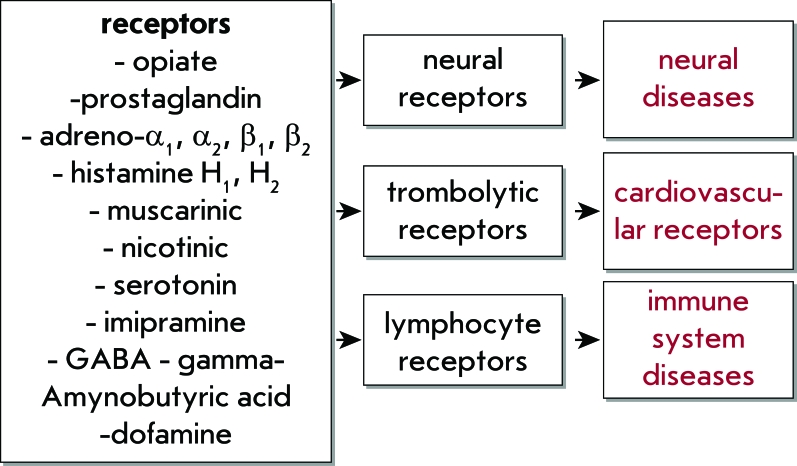
Relation between the receptor portrait of a cell and the features of systemic pathologies (GABA - γ-aminobutyric acid).

Receptors of the cellular membrane can be subdivided into two basic classes: ionotropic and metabotropic. Ionotropic receptors are membrane channels which open or close after binding with a ligand. Emerging ion streams modulate intracellular ion concentrations, which may cause repeat activation of intracellular mediators. Metabotropic receptors may be directly linked to secondary messenger systems. Conformational changes in a receptor during ligand-binding trigger initiation of a biochemical reaction cascade, leading to changes in the functional state of the cell. Membrane receptors play a significant role in intracellular communications, signal transduction into the cell, neural impulse transduction, and many other cell functions. The variability of receptors to various ligand types represented on the membrane of the exact cell constitutes its receptor profile, which determines the type of physiological activity of that cell (Fig. 2).

 It should be noted that the study of the functions of a receptor complex not only allows us to understand the ‘life’ of a normal cell, but can also shed light on the molecular origin of various diseases. On the level of cell receptor system functioning, molecular physiology and molecular medicine are intrinsically linked. P. Erlich’s postulate on the selective effects of curing substances on selective targets (1905) served as the starting point for pharmacological science – in particular pharmacokinetics – in which one of the basic goals is to study the receptor mechanisms of curing substance activity. Receptor profiles determine not only the functional activity of a normal cell, but also the specific pathological conditions of the cells and entire organs. In recent years, many studies have been devoted to both the receptor profile and its dependence on pathological processes in precisely targeted organs. 

**Fig. 3:**
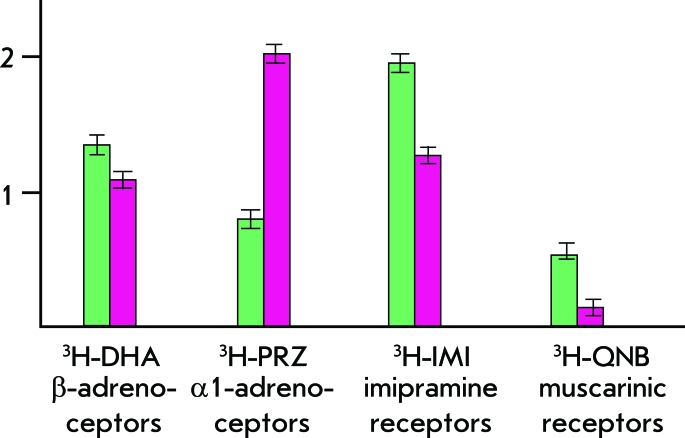
Binding parameters of various receptors of the human lung alveolar macrophages with ligands in normal and chronic inflammations of the lungs ( ^3^ H - tritium, DHA, PRZ, IMI, QNB - studied ligands, green shows the values of Kd for the studied ligands in normal lungs; raspberry - chronic inflammation of the lungs).

We have defined the parameters of the main human alveolar macrophage receptor systems in both normal and pathological conditions [1]. After estimation of the examples of ligand dissociation constants, it was shown that the physiological constant profiles of pathological and normal conditions are substantially different (Fig. 3). We also compared the enzyme activity of phosphorylation systems in normal cells and human tumor cells. It was shown that in normal and tumor cells enzyme activity levels substantially differ; the same tendencies in studies of kinases are observed for different types of tumor cells [2]. An avalanche of experimental data relating to the intracellular “machinery” is being accumulated; specific details of the synapse transduction mechanisms, and knowledge on the structure and functions of normal and defect genes and proteins.

 The significance of the membrane receptor functions and their spatial localization makes these molecules the main targets for the development of drugs against a wide range of diseases, including oncology. In recent years, one of the brightest achievements in molecular physiology has been the development of target drugs based on “therapeutic” monoclonal antibodies. An example of this is trastuzumab, which is based on humanized monoclonal antibodies to receptor ErbB2 (HER2, HER2/neu) of the epidermal growing factor family (EGFR). The drug is widely used for the treatment of such diseases as breast cancer, amongst others. Humanized monoclonal antibodies specifically bind to the extracellular part of the HER2/neu receptor molecule, and they prevent uncontrolled cell proliferation, causing block age of the cell cycle and suppression of angiogenesis. Furthermore, when monoclonal antibodies bind to the target cell, the activation of cell immunity and apparent antibody-dependent cytotoxicity are observed. 

 The implementation of a molecular approach, in this particular case the use of target drugs based on monoclonal antibodies, can considerably improve the clinical situation, improve a patient’s quality of life, and extend their lifespan. Developing target therapeutic drugs being capable of interacting selectively with targets inside the cell or on its surface is one of the priorities in molecular medicine, since the receptor profile of each cell type is unique. This direction is considered to have great potential, since irregularities in the activity of various enzymes or of their regulation is the basic reason behind metabolic disorders and diseases, as enzymes participate in all biochemical reactions and are likely to determine the course of pathological processes. 

Membrane-bound enzymes play a wide range of biological roles, participate in its main processes, such as processing of biologically active molecules, degradation of extracellular matrix components, decomposition or activation of soluble or surface proteins, cell adhesion, and signal transduction into cell. Thus, the activity of different proteases, particularly those localized on the membrane, is believed to be responsible for the events that occur at the early stages of tumor development, because of the disturbances in expression regulation in enzymes belonging to this class [3–6]. We obtained strong evidence of the significance of such an approach in studies of prostate cancer (PC) markers.

Prostate cancer is one of the most widespread tumors in the male population, characterized by a rapid metastasis process [7]. In countries of the European Union, two hundred thousand new cases of prostate cancer are diagnosed annually, with a total of forty thousand deaths occurring [8]. Here, we approach another aspect of molecular physiology and medical intervention – the molecular diagnostics of diseases.

**Fig. 4:**
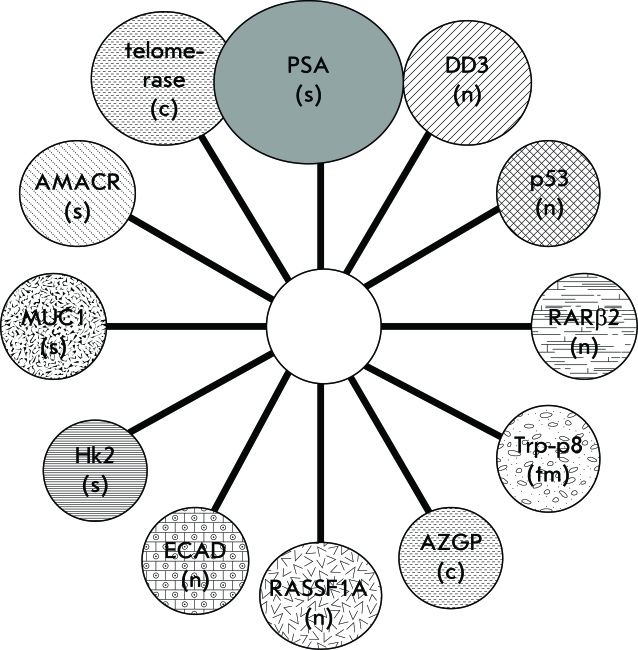
Some markers of prostate cancer and their localization in the cell (PSA - prostate specific antigen, dd3 - differential display code 3, p53 - protein 53, RARβ2 - retinoic acid receptor beta2, Trp-p8 - transient receptor potential-p8, AZGP - zinc a-2-glycoprotein 1, RASSF1A - RAS association domain family protein 1A, ecad - E-cadherin, Hk2 - human prostate specific glandular kallikrein, MUC1 - mucin 1, AMACR - a-methylacyl-coenzyme A Racemase, n - nuclear, c - cytoplasmic, s - soluble, tm - transmembrane).

To this date, a large number of genes and their products which are believed to be involved in the development of PC have been detected, and they can reasonably be considered as markers of this disease [9-14]. Changes in prostate tissue during the malignization process affect all basic cell functions and are reflected on different levels of structures and processes, such as cytomorphological changes, changes in the expression of genes and their products, epigenetic changes, etc. The basic molecular markers, indicating prostate tissue malignization, are illustrated in Fig. 4.

Malignant tumors in the prostate (including PC) can cause changes in the genome, a very significant occurence. In particular, changes appear in the DNA methylation profile [15–24]. Hypermethylation of the 5`-regulatory regions of several genes leads to their inactivation. These changes in genetic material can be used in the diagnosis of prostate pathological conditions.

Amongst the well known epigenetic anomalies is the change in the GST1 gene promoter region methylation profile, found within tumor cells. This gene encodes cytoplasm glutation-S-transferase of the 1 class, which participates in apoptosis regulation and xenobiotic utilization. Hence, in normal cells of the prostate, the promoter region of the GST1 gene is non-methylated, whereas upon proliferative inflammatatory atrophy (PIA) the methylation frequency of this region in the GST1 gene is 6.4%; for highly active prostatic intraepithelial neoplasia (PIN) – 70%; and for prostate adenocarcinoma – 90% [25].

Changes in the methylation status of the 5’-regulatory regions influence not only the GST1 gene, but also genes the products of which participate in tumor proliferation suppression [26-32]. CGI methylation in the promoter regions of such genes causes inactivation, which is associated with increased risk of development of a malignant tumor: RAR2 (retinoic acid receptor 2) gene encodes the protein responsible for the receptor-mediated suppression of tumor growth (retinoids are well-known inhibitors of tumor growth and progress). Methylation of the CpG islands in the promoter region of the RAR2 gene indicates prostate tumor malignization. In healthy pancreatic tissue, methylation of CpG islands is absent [33]. The RASSF1A (RAS association domain family protein 1A) gene is also a tumor growth suppressor. Methylation of the CpG islands in the promoter region of this gene was detected upon malignization of various tissue types [34, 35]. The frequency and methylation rate of RASSF1A correlate with the tumor’s aggressiveness, allowing for an adequate prognosis of the outcome of a disease [36].

 A number of studies performed earlier indicate the key role of DNA damage in the development of the malignization process. The most significant among these damages is considered to be the emergence of multiple chromosome rearrangements and mutations in tumor tissues. Genome instability is a common feature of tumor cells which manifests itself on the level of both chromosomes and selected genes. Moreover, each type of tumor is characterized by an assigned set of the most widespread disorders. In the tumor tissue, a high number of structural rearrangements can be found; firstly, translocations and deletions, the quantity of which significantly rises as the tumor progresses. 

The level of transcripts consisting of the 5’-untranslated region of the androgen-regulated gene TMPRSS2 and exones of the genes of the ETC (ERG4 or ETVI) family is considerably higher in tumor cells [37]. Rearrangements affecting the TMPRSS2 gene and genes of the transcription factors ETC (ERG4, ETVI, ETV4, etc.) occur in prostate tumor cells. These rearrangements result in the generation of chimera oncogenes [38, 39]. Androgen-dependent promoter elements ensure a high level of expression of such chimera oncogenes [38, 39].

 It is necessary to note that there is a positive correlation between the presence of the chimera TMPRSS2-ETC gene transcript and disease severity. A high frequency of detection (50 –60%) is characteristic for mRNA of this chimera gene in prostate adenocarcinoma; whereas for PIN, this value is 16%; and in normal tissue, it is 4%. 

 During the early stages of the disease (when the tumor process is localized), PC is comparably easy to cure; however, as a result of a shortage of diagnosis methods, tumors are usually detected during the final stages of the disease. The current methods are either insufficiently informative or traumatic. Some recent scientific publications reveal losses of some prognostic significance of the basic, presently used biochemical tests for PC – PSA (determination of the prostate-specific antigen concentration in blood). In medical practice false negative and false positive diagnoses based on PSA determination occur frequently. The preliminary diagnosis is confirmed by biopsy; a rather painful procedure with negative consequences for the general condition of the prostate. Thus, the basic goal of a diagnosis is not simply to confirm the disease, but also to reveal the pathogenic process at the earliest possible stage and to determine the stage of the tumor’s progression. 

**Fig. 5:**
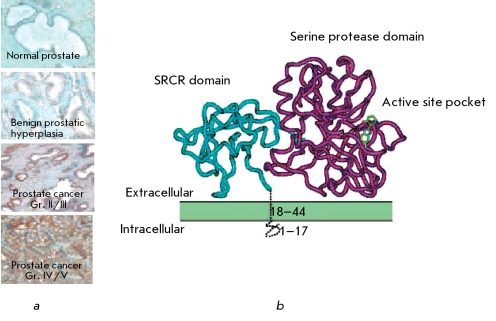
a - Histochemical staining of sections of prostate tissue from patients with prostate diseases of varying severity with monoclonal antibodies against hepsin (color intensity correlates with the expression level of hepsin on the cell surface). b - Domain structure of hepsin and location of the protein relative to the cell membrane (green shows the plasma membrane and the active center of hepsin; blue - SRCR-domain of hepsin; violet - protease domain of hepsin).

Many types of biochemical markers of PC have been described; those originating from serum, urine, semen and prostate tissues. Only a few of these markers can be used in clinical practice, and only one has made it to the clinical trial phase. The markers GSTP1, DD3, AMACR, EPCA, and hepsin are among those showing the most promise. As previously stated, the expression level of hepsin (limited in a normal cell) rapidly increases in a progressing tumor. It is necessary to note that the specificity of high expression levels of hepsin by tumor cells had attracted attention at the earliest stages of the study of that protein. Expression of this enzyme increases as the tumor progresses and reaches a maximum at terminal stages (Fig. 5) [40].

**Fig. 6:**
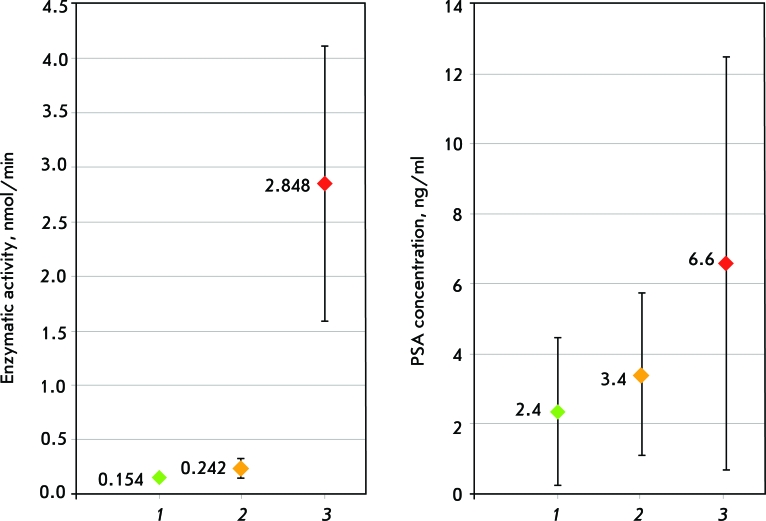
Comparative analysis of diagnostic indexes for diagnostic methods of prostate diseases by determination of the concentration of PSA in the blood of the patient and determination of hepsin activity using “PHOTO-HEPSIN” (the average values of experimental groups and the spread of values). 1 - a group of healthy donors; 2 – a group of BPH patients; and 3 - a group of patients with prostate cancer.

Thus, because of the high specificity of hepsin expression in tumor cells, there is an opportunity to answer the question as to whether the neoplasm in prostate is benign or malignant. Hepsin is preferable to the molecular markers currently being used in clinical practice in terms of several parameters. We propose the following hypothesis: an increase in hepsin proteolytic activity on a cell surface is specific of prostate tumors. For a confirmation of this hypothesis, we obtained a producer strain of recombinant hepsin and found the most specific substrate for it via a process of perfect purification and activation procedures [41]. We proved that it is possible to determine the proteolytic activity in biomaterial samples obtained from males with various pathological conditions of prostate, selected the conditions of this analysis, and we confirmed its specificity in the case of a tumor. The resulting data testifies to the fact that proteolytic activity in a conditionally healthy donor group is similar to that in a group of patients with chronic prostatitis and BPH; but it reliably differed from the proteolytic activity in a group of patients with prostate adenocarcinoma (Fig. 6).

This fact confirms the high specificity of the method: whilst chronic prostatitis and the BPH background level of proteolysis activity are constant (as in conditionally healthy patients), the PC progress level of proteolysis activity rapidly increases, and the basic impact includes hepsin, so far as a specific substrate is used for detection [42].

**Fig. 7:**
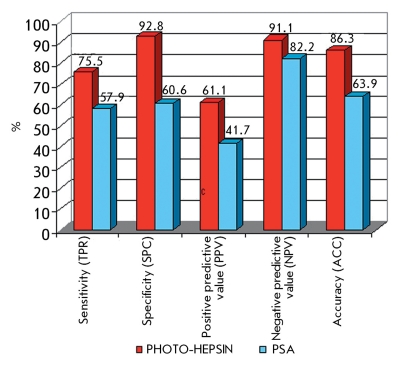
Comparative analysis of the basic evaluative diagnostic indexes of the methods for prostate diseases diagnosis - determination of the concentration of PSA in the blood of the patient and determination of hepsin activity using “PHOTO-HEPSIN” (red shows the values of “PHOTO HEPSIN” parameters; blue shows the values of the parameters of the method for PSA concentration determination; -PV - negative predictive value; and +PV - positive predictive value).

Based on the studies carried out in our laboratory, a PC detection test-kit has been developed. It is based on hepsin activity determination in epithelial cells of the prostate collected with urine after rectal massage (Patent “Prostate cancer detection test-kit and prostate cancer diagnostic method” [Eurasian patent №011694], diagnostic kit (Registration № FSR 2009/05065)). Such parameters as sensitivity, specificity, prognostic significance of positive and negative results, and diagnostic accuracy of the developed method are not inferior to those of the well-known biochemical tests for PC and other methods currently under development; indeed, it proved better by some parameters (Fig. 7).

 Besides, the new method has significant advantages in comparison with the currently widely used PSA concentration determination methods. 

 The method presented by us is better than existing ones, because of the non-invasive character of biomaterial collection; since enzyme activity is measured in urine. To conclude, application of this method, which was developed in conjunction with existing methods, will help to avoid false diagnosis and will have a beneficial impact on the general conditions and quality of life of the patient. 

 The results obtained allow to conclude that the new method of malignant neoplasm of prostate diagnostics with determination of hepsin activity has a set of advantages as compared with the currently widely used PSA determination and can be recommended for PC screening studies in clinical laboratory conditions. 

 Increase of the size of a tumor as a result of increasing, uncontrollable tumor cell proliferation causes them to invade surrounding tissues. This process is followed by the end of any contact both between tumor cells and normal cells; because of the action of various transmembrane proteases, it affects the surrounding territorial matrix, as well. 

Serine protease hepsin is one of the enzymes that regulate the process of local invasion of tumor cells [4]. The expression level of hepsin was shown to considerably increase on the surface of prostate adenocarcinoma cells [43-47]. Hepsin was shown to be the key activation factor of the proteolytic processes in tumor tissue, which result in dissemination of tumor cells. In order to better understand the role of hepsin in these processes, it would help to review how the associated membrane proteolysis participates in the tumor’s progress. During the pathological process, activity of the membrane-bound proteases causes an uncontrollable proteolysis of the territorial matrix and disorganization of its structure.

The majority of studies devoted to evaluating the role of hepsin in oncopathology have focused on tumors of the prostate. We shall review the basal membrane disorder of this organ from the perspective of molecular physiology and the potential role of hepsin. The basal membrane of the prostate is a specialized extracellular structure separating epithelial and stromal cells from each other and consisting of the matrix proteins produced by these two cell types [48]. Disruption of this structure is necessary for a local invasion in the early metastasis process [49].The molecular mechanisms involving hepsin in the progress of malignant neoplasm has become clear. The set of extracellular matrix components which are potential hepsin substrates have been discovered [4, 50].

**Fig. 8:**
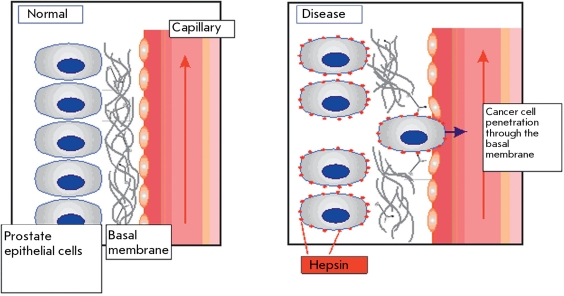
Invasion of tumor cells through the basement membrane with the participation of hepsin.

Another aspect of hepsin’s impacts on the pathological process is the activation of enzymes in inactive form that are also involved in the process of basal membrane degradation [51]. Thus, hepsin can multiply and increase proteolysis on the surface of tumor and stromal cells, which aggravates the damage to the basal membrane and accelerates tumor progress (Fig. 8).

This hypothesis confirms the data on the suppression of the tumor cell’s invasive growth when hepsin activity is inhibited [52]. This, and other features of hepsin, makes it a convenient target for therapeutic actions. Therefore, studying the inhibition mechanisms is considered promising in the development of antitumor drugs that can be effective in case of tumors for which an elevated expression of hepsin is characteristic. The search for specific hepsin inhibitors is underway, with the purpose of creating targeted drugs and developing PC therapy methods [53]. Hepsin is involved in such phenomena as the increase in cell motility, matrix protein separation and extracellular structure disorganization, and activation of extracellular proteases and their cascades, which underlie tumor progression. Inhibition of the activity of this enzyme may lead to suppression of these processes, and it will positively influence disease outcome.

In 2008, in a screening of the libraries of drugs and various chemical compounds aimed at searching for potential low-molecular-weight inhibitors of hepsin, a set of compounds capable of specifically inhibiting its proteolytic activity were found. Anthralin (anthracene-1,8,9-triol) emerged as one of the most efficient inhibitors of hepsin. Some compounds were dose-dependently shown to suppress the activity of recombinant hepsin and to exhibit no cytotoxic effect on various cell lines, which is significant for therapeutic applications. Among all compounds, anthralin demonstrated the highest inhibiting ability towards hepsin: it inhibited hepsin 5.5 and 85 times more efficiently than trypsin and thrombin, respectively [53].

**Fig. 9:**
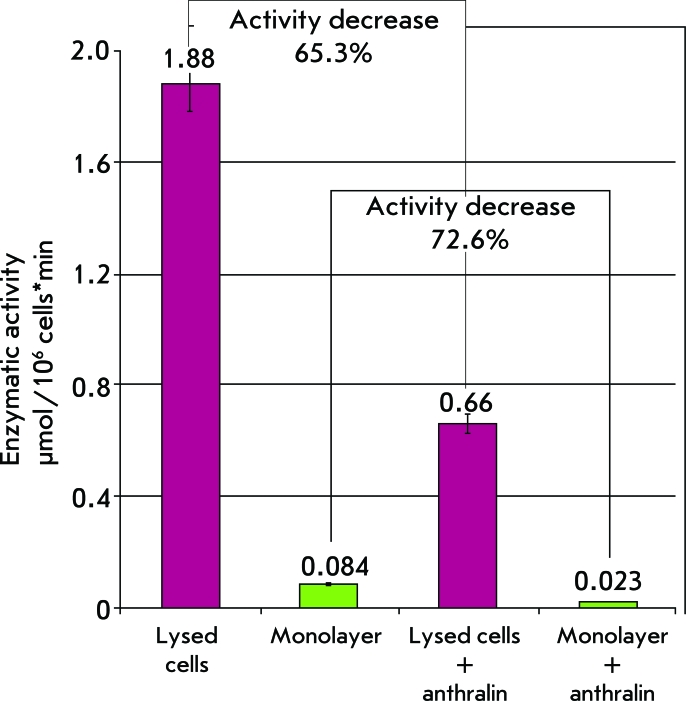
Comparison of the proteolytic activity of live and lysed LnCap cells in the absence and presence of anthralin (purple shows the data for lysed cells, green shows the data for a monolayer of living cells).

As was discovered earlier in our laboratory, anthralin inhibits recombinant hepsin. Due to this, we assumed that anthralin may affect the native form of the protein in the same way [41]. The main contribution to proteolytic activity on the surface of prostate adenocarcinoma cells is made by hepsin, since it is its gene that is over-expressed. Therefore, it can be assumed that the impact of a specific hepsin inhibitor will considerably decrease the general proteolytic activity. The introduction of Anthralin into lysated human prostate adenocarcinoma cells caused a 50–70% reduction in the general proteolytic activity, which attests to the fact that anthralin has an efficient inhibiting action on native hepsin localized on the membranes of tumor cells. Fig. 9 shows the results of a determination of the proteolytic activity of a LnCap human prostate adenocarcinoma cell line in the presence and absence of anthralin.

 The impact on the specified enzyme systems can control the disorders observed, while membrane localization of a number of enzymes is extremely convenient for designing targeted drugs and their application. The controlled enzyme activity typically underlies the approaches that are applied for the treatment of various diseases and the development of new drugs. With account for the abundance of serine proteases and their participation in pathological processes, the search for new inhibitors is of great significance. Even today, some compounds capable of suppressing protease activity are used in tumor therapy. Data confirming elevated expression of some members of the family of transmembrane serine proteases upon various oncopathologies has been published. The inhibition of the activity of enzymes belonging to this family is considered as promising in antitumor therapy. 

 Transmembrane localization of hepsin makes this enzyme a good target for therapeutic agents, since the localization of the transmembrane domain of this protein on a cell’s surface may facilitate drug delivery. Another advantage of hepsin when used as a molecular target is that the negative effects in hepsin suppression would be minimal, whereas the high specificity of its expression by malignant tissue cells can be used for effective and specific antitumor therapy. 

 In the present paper, we have attempted to summarize the main features of molecular physiology as a new interdisciplinary field of fundamental knowledge on sophisticated biological systems. Molecular physiology has a special place in the variety of contemporary life sciences. This role has to do with the connection of molecular physiology to medicine and the stupendous number of potential medical applications. Furthermore, the role is associated with the conceptual revolution which has been taking place over the past 10–15 years. These factors allow to regard molecular physiology as a common discipline in biology which is located at the border between such sciences as biochemistry, bioorganic chemistry, molecular and cell biology, microbiology, and evolutionary biology. 
